# Assessing the Diagnostic Accuracy of Teledermatology Consultations at a Local Veterans Affairs Dermatology Clinic

**DOI:** 10.7759/cureus.15406

**Published:** 2021-06-02

**Authors:** Caroline A Gerhardt, Rachel Foels, Stefanie Grewe, Brooke T Baldwin

**Affiliations:** 1 Medicine, University of South Florida Morsani College of Medicine, Tampa, USA; 2 Internal Medicine, Lehigh Valley Health Network, Allentown, USA; 3 Pathology, University of South Florida Morsani College of Medicine, Tampa, USA; 4 Dermatology, James A. Haley Veterans' Hospital, Tampa, USA

**Keywords:** teledermatology, telemedicine, virtual care, dermatology diagnostic concordance, diagnostic concordance, online dermatology

## Abstract

With the advances in health information technology and the need for increased access to specialized health care, the advent of telemedicine was designed to bring care to individuals at a distance. Telemedicine decreases barriers to health care and brings medical specialists to underserved areas and populations. We have seen a tremendous increase in the need and utilization of telemedicine during the COVID-19 pandemic due to the lockdowns and social distancing efforts. Virtual care continues to be extended to patients to maintain their health care needs when in-person clinic appointments are not feasible or ideal such as seen during a pandemic. Telemedicine is an additional tool that has proven vital to our healthcare system. To provide optimal care, a strong technological infrastructure must be in place. Once in practice, positive outcomes have been noted for patients and healthcare providers as diagnosis, treatment, and appropriate triage can be made virtually and at the patients’ convenience. To ensure high-quality care is provided through the Veterans Affairs teledermatology consultation service, we investigated the concordance of teledermatology diagnoses with clinical examination findings through a retrospective chart review covering a one-year time period. Our study found a concordance of 75.3% between the teledermatology diagnoses and the in-person clinical diagnoses. The main limitation we found to virtual examination is the inability to perform total body skin examinations. We found that 60.2% of patients had additional diagnoses when examined in person, with 8.4% of patients having an additional malignant diagnosis. These findings highlight the need for in-person examinations when feasible to ensure that no other diagnoses go undiscovered if not captured on the submitted images for teledermatology consultation. Despite the limitations posed by photographic examination, teledermatology can be used as a reliable method for diagnosis when a conventional in-person examination is not readily available or ideal, such as during a pandemic, and can serve as a powerful triaging tool.

## Introduction

The utilization of technology in the healthcare system has become increasingly important in the digital era. As the shortage of physicians continues to increase, we must expand the boundaries of health care to meet our diverse and growing population needs. Some of the proposed benefits of telemedicine include increased efficiency, increased access to care for individuals in rural areas, increased access to specialty care, decreased costs, decreased need to travel, and triaging for in-person (IP) visits [1].

During the coronavirus disease (COVID-19) pandemic, we have seen a surge in telemedicine and virtual care utilization due to government-mandated lockdowns and social distancing efforts [2]. With the generalizable acceptance and satisfaction surrounding telemedicine, it is likely to be increasingly utilized and expanded after the COVID-19 pandemic. Dermatology is among the many fields that utilize telemedicine technology to bring skin examinations to patients from the comfort and safety of their homes.

Teledermatology (TD) is a method of care in dermatology that is practiced at a distance by employing telecommunication technologies such as photography and videography. These modalities are the two distinct common forms of TD that are typically used in the healthcare setting. The store and forward (SAF) technique captures a photo of the lesion of concern, which is then sent directly to the dermatologist to be reviewed, diagnosed, and triaged. The live interactive (LI) technique utilizes live video interaction with the patient and dermatologist in real-time.

The literature has demonstrated a strong diagnostic concordance between TD and IP evaluations. Studies comparing the SAF technique with IP evaluations demonstrate a concordance ranging from 54% to 91% [3-10]. Despite this large range of concordance, these studies elucidate the potential for strong diagnostic accuracy when using TD. Furthermore, studies have consistently demonstrated both improved patient and physician satisfaction with TD [11-13]. In a study conducted during the COVID-19 pandemic, the most common reasons cited for supporting TD included time efficiency, not requiring transportation, and maintaining social distancing [12].

Our clinic, which serves a veteran population, has utilized TD for several years. Before the COVID-19 pandemic, it had been implemented to serve our patient population better and increase access to care. Many of our veterans reside a great distance from our clinic and/or do not have access to reliable transportation. TD helps to better serve these patients by effectively triaging patients and avoiding unnecessary IP visits. Our platform utilizes the SAF technique. Our dermatologists can triage the lesion of concern, manage it remotely, and/or recommend IP follow-up as appropriate. Triaging allows patients with more severe dermatological concerns to be prioritized with IP visits, while those with less concerning lesions may benefit from easier access to care from home.

While there are numerous benefits to employing telemedicine, it is essential to ensure quality management. Our study looks at the concordance between the TD diagnoses and the IP diagnoses to ensure we are meeting our goals of providing the highest quality care to our veteran population. We also explore the limitations of TD.

## Materials and methods

In our study, we performed a retrospective chart review to assess diagnostic concordance between our TD clinic and IP follow-up visits. We reviewed charts over a one-year period from January 2017 to December 2017. Over this time period, our TD service diagnosed and managed 1,286 patients. Inclusion criteria included patients seen both by TD and IP follow-up. Patients who were not seen at an IP visit were excluded. Variables collected during chart review included: date of consultation request, date of service, diagnosis rendered, management recommended, clinical diagnosis made on follow-up examination, and any additional findings. Our primary objective was to determine the diagnostic concordance between the initial TD diagnosis and diagnosis rendered at IP follow-up. This was calculated as a percent agreement between the two diagnoses. Secondary objectives include (1) determining the percentage of patients who were found to have additional diagnoses at IP follow up and (2) determining the percentage of patients who were found to have an additional malignant lesion at IP follow up.

## Results

**Figure 1:**
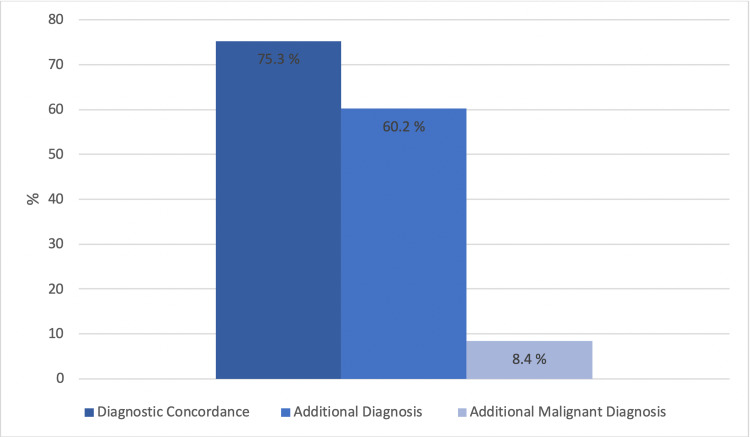
Diagnostic concordance of teledermatology diagnosis with in-person diagnosis, percentage of patients with additional diagnoses at in-person visit, and percentage of patients with additional malignant diagnosis discovered on in-person visit.

Out of the 1,286 patients seen by TD, 809 were recommended to subsequently follow up at an IP visit. There were 477 patients of which the teledermatologist was confident in treating the patient exclusively via TD and therefore did not recommend follow-up IP. The mean time from TD visit to IP clinic visit was 30.4 days. The complete concordance between the SAF TD diagnoses and the IP diagnoses was 75.3% (Table 1 and Figure 1). Of the 809 patients seen at an IP follow-up visit, 60.2% had at least one additional diagnosis. Furthermore, 8.4% of the patients seen at an IP follow-up visit had an additional lesion that was diagnosed as malignant, 1.1% being a malignant melanoma.

**Table 1:** Results showing the total number of patients seen via teledermatology, number of patients seen in-person for a follow-up visit, diagnostic concordance between the teledermatology diagnosis and in-person clinic diagnosis, number of additional diagnoses made at the in-person clinic visit, number of additional malignant diagnoses made at the in-person clinic visit, and number of additional malignant melanoma diagnoses made at the in-person clinic visit 95% CI = 95% Confidence Interval

	Number of Patients	%	95% CI
Total Seen by Teledermatology	1286	100	
Follow-Up in Clinic	809	62.9	(60.2-65.5)
Complete Diagnostic Concordance	609	75.3	(72.2-78.2)
Additional Diagnosis in Clinic	487	60.2	(56.7-63.6)
Malignant Diagnosis in Clinic	68	8.4	(6.6-10.5)
Malignant Melanoma Diagnosis in Clinic	9	1.1	(0.6-1.8)

## Discussion

Periodic evaluation of clinical services is important in all healthcare settings to ensure that the intended goals and missions are met. We evaluated our TD consultation service by determining the concordance of diagnosis in those evaluated via TD and IP follow-up to ensure our service provided optimal healthcare to our veteran population.

In the patients with a TD visit followed by an IP visit, the diagnostic concordance was 75.3%. This is relatively consistent with previously reported concordance values that compare SAF diagnoses with follow-up IP diagnoses [4-10]. Two dermatologists at our site were utilizing TD during the period analyzed for our study. The diagnostic concordance rate for the first dermatologist was 76.3%. This dermatologist saw 81.5% of the patients included in the study. The diagnostic concordance rate for the second dermatologist was 68.6%. A systematic review conducted by Warshaw et al. calculated a mean weighted diagnostic concordance for 10 studies that assessed the SAF technique [14]. The total number of combined patients was 1,077, and the aggregated diagnostic concordance was 65.3% [14]. Our study further adds to the evidence that various skin lesions can be accurately diagnosed and treated via TD.

A limitation to our study is that not every patient evaluated by TD was subsequently seen in the clinic. When the dermatologist was confident in a benign diagnosis through TD, the patient was not advised to follow up at an IP visit. Since the patients with unambiguous diagnoses through TD were excluded from the calculation, it is possible that our true concordance may be higher than what is reported here.

Another critical component for the success of TD is the quality of the images sent to the dermatologist for review. This study included patients regardless of the quality of the images received to better account for what would be expected in reality. When the image quality was poor, the patient was more likely to have to follow-up IP. Additional barriers to accessing TD include poor technological literacy, not having a smartphone or camera, and unreliable internet connection.

Perhaps the greatest shortcoming of TD is the inability to perform a total body skin examination. Sixty percent of the patients who followed up at an IP visit had at least one additional diagnosis. Of particular concern is the possibility of overlooking a malignant lesion, as 8.4% of the patients were found to have a malignant lesion at a secondary site during their IP follow-up. Therefore, it is important to acknowledge that TD should not be used as a substitute for total body skin examinations. Despite these limitations, TD is a powerful tool for triaging patients and an adjunctive means to address acute skin concerns.

## Conclusions

In our study, we aimed to ensure that our intended mission to increase access to care, while maintaining the highest level of patient care standards, was being achieved. Based on our findings we endorse TD consultation, via high-quality images sent in real-time, for specialist evaluation. TD can be a reliable method for diagnosis when an IP conventional examination is not readily available or feasible. While there are limitations to virtual examination including, image confines and clarity, limited history communication, and the lack of tactile examination, most lesions and rashes can be evaluated with the use of high-quality media images. When faced with these limitations, a conventional examination is recommended for further clarification.
